# Mesothelioma and Colorectal Cancer: Report of Four Cases with Synchronous and Metachronous Presentation

**DOI:** 10.3390/ijms23052630

**Published:** 2022-02-27

**Authors:** Gabriella Serio, Federica Pezzuto, Francesco Fortarezza, Andrea Marzullo, Maria Celeste Delfino, Antonio d’Amati, Daniele Egidio Romano, Sonia Maniglio, Concetta Caporusso, Teresa Lettini, Domenica Cavone, Luigi Vimercati

**Affiliations:** 1 Department of Emergency and Organ Transplantation—DETO, Pathology Unit, University of Bari, G. Cesare 1 Sq., 70121 Bari, Italy; gabriella.serio1@uniba.it (G.S.); andrea.marzullo@uniba.it (A.M.); damatiantonio@yahoo.it (A.d.); danieleromano95@gmail.com (D.E.R.); sonya1996@hotmail.it (S.M.); kcaporusso.c@libero.it (C.C.); lettinit@yahoo.com (T.L.); 2 Department of Cardiac, Thoracic, Vascular Sciences and Public Health—DCTV, Pathology Unit, University of Padova, 61 N. A. Gabelli St., 35121 Padova, Italy; federica.pezzuto@unipd.it; 3 Pathology Unit, University Hospital of Padova, University of Padova, 61 N. A. Gabelli St., 35121 Padova, Italy; 4 Department of Interdisciplinary Medicine, Occupational Health Division, Internal Medicine Unit, University of Bari, G. Cesare 1 Sq., 70121 Bari, Italy; maria.delfino@uniba.it (M.C.D.); domenica.cavone@uniba.it (D.C.); luigi.vimercati@uniba.it (L.V.)

**Keywords:** asbestos, mesothelioma, peritoneum, pleura, colon cancer, *BAP1*, *CDKN2A*

## Abstract

There is evidence that asbestos could play a role in the carcinogenesis of digestive cancers. The presence of asbestos fibres in histological samples from gastric, biliary, colon cancers has been reported, but the mechanism is still controversial. It has been hypothesised that asbestos reaches these sites, especially through contaminated water; however, some experimental studies have shown that the inhaled fibres are mobile, so they can migrate to many organs, directly or via blood and lymph flow. We report four unusual cases of colorectal cancers in patients with a long history of asbestos exposure who also developed synchronous or metachronous mesothelioma. We evaluated the roles of BRCA associated protein-1 (*BAP1*) and cyclin-dependent kinase inhibitor 2A (*CDKN2A*) in colon cancer and mesothelioma to support the hypothesis that *BAP-1* and *CDKN2A* are tumour suppressor genes involved in disease progression, recurrence, or death in both digestive cancers and mesothelioma. Potentially, these markers may be used as predictors of worse prognosis, but we also stress the importance of clinical surveillance of exposed patients because asbestos could induce cancer in any organ.

## 1. Introduction

The carcinogenicity of asbestos is known, and the relationships between asbestos and numerous human cancers (laryngeal, ovarian, testis, prostate, and bladder cancers) have been reported [1,2,3,4,5,6,7,8]. Furthermore, much evidence supports the association between asbestos exposure alone or in synergy with other risk factors (i.e., radiation or smoking) and colon cancer [9,10,11,12].

However, aspects concerning the mode of absorption (respiratory inhalation or oral ingestion of contaminated water), the time of exposure, the type of exposure (occupational or environmental), the concentration, and the relationship between the different asbestos fibres and cancers of other sites have not been clarified [13]. The known mechanisms of asbestos carcinogenesis are chronic inflammation (including frustrated phagocytosis) and loss of tumour-suppressive mechanisms. Although several genetic alterations have been reported, the most frequent chromosomal aberrations in pleural and peritoneal mesotheliomas are loss at 3p21 (*BRCA1* associated protein-1—*BAP1*) and 9p21 (cyclin-dependent kinase inhibitor 2A—*CDKN2A*). *CDKN2A* is a tumour suppressor gene that prevents uncontrolled cell proliferation by initiating cell cycle arrest and apoptosis. Homozygous *CDKN2A* deletion is more frequent in pleural mesothelioma, represents a good diagnostic marker and seems to be related to a poor prognosis. BAP1 is a nuclear-localised deubiquitinating enzyme involved in many processes, including DNA damage response and regulation of the cell cycle and cell proliferation [7,14,15,16,17,18].

Colon cancer represent the third most frequent malignant disease in terms of incidence and mortality worldwide [19]. Some epidemiological observations suggest an increased risk of colorectal cancer in asbestos-exposed patients [9,10,14,19,20], even if the molecular mechanisms have not yet been investigated and clarified. Moreover, according to the recent consensus statement on asbestos-related neoplasms, colorectal cancers cannot yet be considered as asbestos-related diseases [21,22].

The aim of the present study was to report four cases of colon cancer arising in asbestos-exposed patients, two of whom were affected by synchronous mesothelioma and two of whom developed mesotheliomas (metachronous) four years later with the analysis of the molecular status of *CDKN2A* and BAP1 expression in all tumours.

## 2. Results

**Figure 1:**
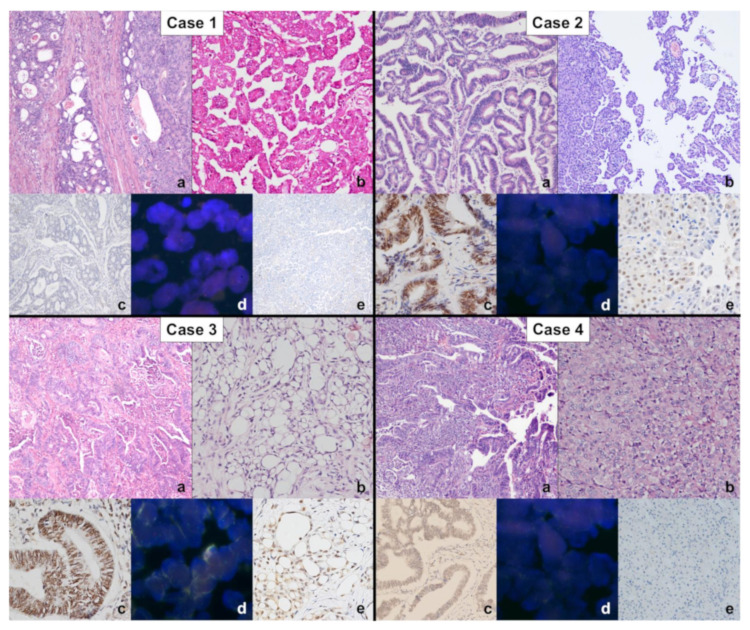
Each box groups the main histological, immunohistochemical, and molecular images of both colon cancer and mesothelioma of each patient as follows: histological microphotographs of colon adenocarcinomas (**a**), haematoxylin and eosin stain (original magnification ×100) and mesotheliomas (**b**), haematoxylin and eosin stain, original magnification ×100 (cases 1, 2, and 3), ×200 (Case 4); immunohistochemistry for BAP1 in colon adenocarcinomas (**c**), original magnification ×100 (case 1 and 4), ×200 (cases 2 and 3); FISH analysis for *CDKN2A* (p16) in colon cancer samples (**d**); immunohistochemistry for BAP1 in mesotheliomas (**e**), original magnification ×100 (cases 1, 3, and 4), ×200 (case 2). The pathological, immunohistochemical, and molecular findings are described in Table 2.

**Figure 2:**
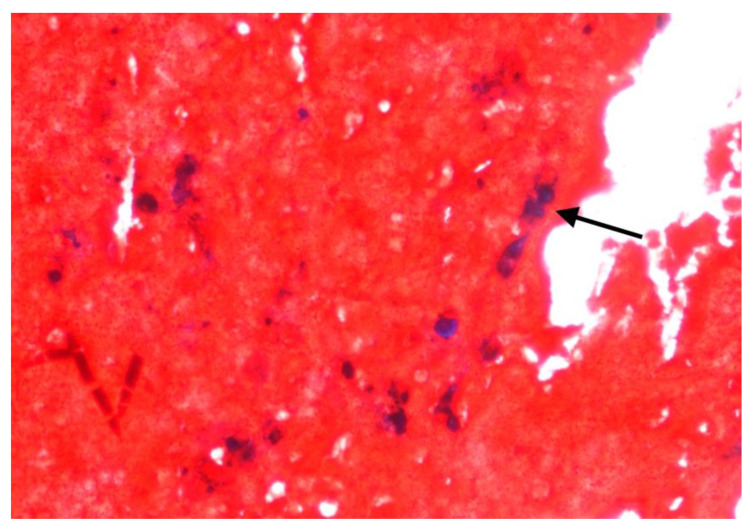
Cytoblock of the ascitic fluid of patient 1. An asbestos body is clearly visible (arrow) (Perls’ Prussian Blue stain, original magnification ×400).

Clinicopathological data, survival, asbestos exposure time, family history of cancer, BAP1 immunohistochemistry, and *CDKN2A* FISH results are shown in Table 1 and Table 2. The main histological, immunohistochemical, and molecular images of each patient are grouped in Figure 1. No asbestos fibres were detected in any of the colorectal adenocarcinoma samples. However, asbestos bodies were identified in in the ascitic fluid cellblock of the patients 1 (Figure 2).

The complete absence of BAP1 expression was found in two patients, both in intestinal tumours and in mesotheliomas.

All tumours had *CDKN2A* deletions above the cut-off, heterozygous or homozygous. In particular, intestinal tumours showed higher values of heterozygous deletions than homozygous ones (median value 63% vs. 29%, respectively) while mesotheliomas showed a completely opposite scenario with higher percentages of homozygous deletions (median value 16% vs. 72.5%).

In situ and molecular biomarker testing for colorectal cancer did not detect any mutations. Three patients (1, 2, 3) received adjuvant chemotherapy (FOLFOX, 5-FU/LV + oxaliplatin) for colon cancer; one patient (case 4) received only palliative chemotherapy due to poor performance status. Patients with metachronous mesothelioma received palliative chemotherapy (cisplatin + pemetrexed or gemcitabine), and patient 1 received treatment with cisplatin after FOLFOX chemotherapy.

## 3. Discussion

Our study is the first in the literature to analyse the expression of BAP1 and the deletion of *CDKN2A* in four colon adenocarcinomas with synchronous or metachronous mesothelioma of patients with well documented occupational and environmental exposure. Molecular analysis for *CDKN2A* documented the deletion of the gene in all eight tumours, with a prevalence of heterozygous deletion in the colorectal cancers and homozygous one in mesotheliomas. Moreover, in two cases both mesothelioma and intestinal carcinoma showed loss of BAP1 expression.

An increased risk of asbestos-related tumours appears evident owing to respiratory exposure, but the risk of cancer development after oral ingestion of asbestos from drinking asbestos-contaminated water remains unclear. Although inhaled asbestos has undoubtedly greater toxicity in the lung and therefore in the pleura, it has been shown that, through blood or lymphatic flow, fibres can translocate to other organs [23]. In support of the first observations reported by Selikof et al. [24], experimental studies have demonstrated the passage of chrysotile and crocidolite fibres across the gastrointestinal wall, suggesting the hypothesis of fibre dissemination, inhaled or ingested, through the blood and/or lymphatic flow [25]. Auerbach et al. [26], in a series of 19 autopsy cases, observed the presence of asbestos fibres or ferruginous bodies not only in the lung but also in other organs. In our case series, we histologically identified an asbestos body in the ascitic fluid of a patient with pleural mesothelioma and colon adenocarcinoma even if microanalysis studies would be desirable to better deepen the research. The effects of oral ingestion of asbestos-contaminated water are discordant between studies; in fact, whereas many studies did not reveal an excess of cancer mortality after oral asbestos ingestion, other studies report a clear dose–response relationship between different asbestos fibres and digestive cancers [27,28]. Clin et al. [29] and Paris et al. [10] observed a significant increase in death in patients who had developed oesophageal or colon cancers and who had a documented history of asbestos exposure. The presence of asbestos fibres in the colon or other viscera cannot be considered accidental but is related to the development of the disease. Therefore, the association between colon cancer that represents the third most frequent malignant disease in terms of incidence and mortality worldwide in workers or in subjects otherwise exposed to asbestos appears consistent. Similarly to our cases, there is some evidence that in workers, the inhalation of asbestos increases the risk of colorectal cancer [30] but these often-stringent epidemiological observations are limited and/or not consistent due to the absence of molecular markers supporting the development and progression of cancer into sites other than the mesothelium. The collection of these cases to reach an adequate number of evidence to support statistical correlations would be desirable.

From a molecular point of view, many colorectal cancers show chromosomal instability, characterised by gains or losses of numerous chromosomal regions that drive the process from neoplastic growth to invasiveness and metastasis development [19]. Some studies reported an increased risk of colorectal cancer in patients with loss of heterozygosity at the *BRCA1* gene locus, which shows shorter disease-free survival [31,32]. Mutations of *BAP1* have been described in multiple human cancers, and their role often depends on the site of the tumour and the triggering factors. Regarding our case-series, BAP1 was not expressed in two cases and, interestingly, in both mesotheliomas and intestinal adenocarcinomas. This feature raises the suspicion of the presence of a *BAP1* germline mutation, also because the family history of these two patients showed many more cases of neoplasia than the other two. This would have been an important aspect to investigate in our case studies but unfortunately it was not possible to evaluate the possible germline mutation of *BAP1* in the described cases for the lack of consent to genetic analyses. Germline and somatic *BAP1* inactivation predispose patients to a higher risk of both familial and sporadic mesothelioma. Patients with germline *BAP1* mutations seem to have better survival than those with somatic alterations [33]. The role of *BAP1* in colorectal cancer is unclear and poorly investigated. Tang et al. [34] observed that *BAP1* is downregulated in colon cancer and associated with shorter survival. It would be of interest to study this marker in this direction to assess its prognostic role in tumours other than mesothelioma.

Another interesting finding in our study was that *CDKN2A* deletion was present in all cases, in particular homozygous deletions in mesotheliomas and heterozygous deletions in intestinal neoplasms. Deletion of *CDKN2A* is frequent in mesothelioma and its prognostic implications have been described [35,36]. Losses or inactivation of *CDKN2A* have also been observed in colon cancer and seem to be related to tumour progression and prognosis. Berg et al. [37], analysing a series of colon cancers, observed that higher copy number alterations and losses at 9p21 were significantly related to advanced stage and shorter survival. Although the number of cases is limited and the comparison of survivals is inapplicable, our case series enriches this evidence. Moreover, the combination with the lack of BAP1 expression and the onset of mesothelioma in the same patients may lead us to speculate on a possible common pathway of carcinogenesis linked to asbestos.

The intrinsic limitations of our study, such as the retrospectivity of the study design and the limited number of cases, make this result ambitious and future study necessary. Nevertheless, the rarity of mesothelioma itself and its co-occurrence with other neoplasms give value to the report. The molecular investigations we have performed were limited to specific targets. Other genes could be involved by biallelic inactivation, such as *NF2* [38], typically lost for monosomy or large deletion of chromosome 22. This aspect brings out another necessity, namely the employment of more sophisticated sequencing techniques, capable to detect other mutations and small fragment deletion. Lastly, to strengthen the link between asbestos and colorectal tumours, the search for asbestos fibres in intestinal cancer samples should be performed with electron microscopy coupled with microanalysis. This, together with the collection of more cases that will be analysed based on next-generation or other high-throughput sequencing methodologies, will represent the goal of future communications.

## 4. Materials and Methods

The present study was conducted in accordance with local ethics regulations and the Helsinki Declaration. The medical history and clinicopathological information of each patient were obtained from treatment records. Patients’ asbestos exposure (occupational and/or environmental), clinical data (sex, age, and stage of the disease), date of diagnosis, and survival were collected through Apulia regional mesothelioma register questionnaires, classified according to the National Mesothelioma Register (ReNaM) criteria (https://www.inail.it/cs/internet/docs/renamlineeguida2005-pdf.pdf; accessed on 24 February 2022), and encoded in an electronic database.

Colon cancer symptoms were the first clinical manifestation; all patients underwent surgery, and none of them received chemotherapy and/or radiotherapy prior to the surgery. After radical resection, all pathological tissue specimens were formalin-fixed and paraffin-embedded. Haematoxylin–eosin-stained sections were reviewed by two expert pathologists to confirm the diagnosis of colon and mesothelioma synchronous tumours (two cases). For colon cancer diagnosis, the WHO [19] criteria were applied, and for mesothelioma, a wide panel of immunohistochemical antibodies was used, following WHO recommendations [7]. In situ and molecular biomarker testing for colorectal cancer (including mismatch repair protein expression and characterisation of RAS mutations) was also performed. Asbestos fibres were searched in histological specimens of colorectal carcinomas and in an available case of cytoblock of ascitic fluid (Patient 1) using Perls’ Prussian Blue stain. The mesothelioma samples were collected from thoracoscopic pleurectomies and omental and peritoneal biopsies.

Colon cancer and mesothelioma were also immunostained for a BAP1 (clone C4, Santa Cruz Biotechnology Inc., dilution 1:400) antibody. BAP1 nuclear expression was scored as negative (0–10%), low (11–25%), moderate (26–50%), or strong (>50%) in colon cancer and mesothelioma, as suggested by Tang et al. [32], independent of nuclear intensity.

The status of the *CDKN2A* gene was determined by fluorescence in situ hybridisation (FISH). A FISH locus specific *CDKN2A* (9p21) probe (Abbott, Abbott Park, IL, USA) was used to detect chromosome 9 deletion. Vysis LSI *CDKN2A*/CEP 9 probes are provided in one vial as a mixture of the LSI *CDKN2A* (p16) probe labelled with Spectrum Orange and the CEP 9 probe labelled with Spectrum Green. The LSI *CDKN2A* probe spans approximately 222 kb and contains several genetic loci, including D9S1749, DS1747, p16 (INK4B), p14 (ARF), D9S1748, p15 (INK4B), and D9S1752. The CEP 9 Spectrum Green probe hybridizes to alpha satellite sequences specific to chromosome 9, CE Marked. At least 100 cells were scored for each case. A cut-off of 20% was used for homozygous deletion. Heterozygous deletion was defined as when >20% of the cells showed only one signal or a lower signal number than CEP9.

## 5. Conclusions

Occupational/environmental asbestos exposure deserves continued attention because this mineral defined as a “hidden killer” can reach any organ, and the long latency that characterizes the onset of the tumour could obscure the causal relationship. In tumours arising in asbestos-exposed patients, a further molecular analysis is warranted to evaluate the causality between asbestos exposure and carcinogenesis as an alternative to the most common altered pathways.

## Figures and Tables

**Table 1:** Clinical data of patients with colorectal cancer and mesothelioma.

Patient	Sex (M/F)	Age (Years)	Asbestos Exposure	Overall Survival (Months)	Family Cancer History
1	M	70	1958–1989 (occupational, merchant marine deck officer)	51	Father lung cancer Sibling lung cancer Sibling hepatocarcinoma Sibling colon cancer
2	F	58	1971–1975 and 1980–1990 (occupational, dressmaker)	74 (alive)	Father laryngeal cancer
3	M	71	1960–1980 (occupational, carpenter)	42	None
4	M	89	1957–1975 (occupational, welder) 1971–2018 (environmental, resident near asbestos factory)	5	Father colon cancer Sibling bone tumour Sibling pancreatic carcinoma

**Table 2:** Pathological and molecular data of patients with synchronous and metachronous mesothelioma.

Patient	Tumour	Site	pTNM	BAP1 (IHC)	*CDKN2A* Homozygous Deletion	*CDKN2A* Heterozygous Deletion	Therapy
1 *	High grade adenocarcinoma, NOS	Colon	T2N0M0	Score 0 (0%)	32%	50%	FOLFOX
2 **	Low grade adenocarcinoma, NOS	Rectum	T2N1cM0	Score 4 (90%)	28%	62%	FOLFOX
3 **	Low grade adenocarcinoma, NOS	Rectum	T3N2aM0	Score 4 (78%)	23%	64%	FOLFOX
4 *	Low grade adenocarcinoma, NOS	Rectum	T3N0M0	Score 0 (10%)	30%	73%	-
1 *	Epithelioid mesothelioma	Pleura	-	Score 0 (0%)	70%	12%	Palliative
2 **	Epithelioid mesothelioma	Pleura	-	Score 3 (60%)	44%	54%	Palliative
3 **	Biphasic (Epithelioid adenomatoid/solid 80%) mesothelioma	Peritoneum	-	Score 3 (55%)	90%	10%	Palliative
4 *	Biphasic (Epithelioid solid/trabecular 70%) mesothelioma	Peritoneum	-	Score 0 (0%)	75%	20%	Palliative

Legend: * Synchronous and ** metachronous colorectal cancer and mesothelioma; IHC: immunohistochemistry; TNM classification sec. WHO 2018; FOLFOX: 5-FU/LV+ Oxaliplatin.

## Data Availability

Data sharing is not applicable to this article.
